# Dynamic evolution of V1R putative pheromone receptors between Mus musculus and Mus spretus

**DOI:** 10.1186/1471-2164-10-74

**Published:** 2009-02-09

**Authors:** Vanessa C Kurzweil, Mike Getman, NISC Comparative Sequencing Program, Eric D Green, Robert P Lane

**Affiliations:** 1 Department of Molecular Biology and Biochemistry, Wesleyan University, Middletown, CT 06457, USA; 2 Genome Technology Branch and NIH Intramural Sequencing Center (NISC), National Human Genome Research Institute, National Institutes of Health, Bethesda, MD 20892, USA

## Abstract

**Background:**

The mammalian vomeronasal organ (VNO) expresses two G-protein coupled receptor gene families that mediate pheromone responses, the V1R and V2R receptor genes. In rodents, there are ~150 V1R genes comprising 12 subfamilies organized in gene clusters at multiple chromosomal locations. Previously, we showed that several of these subfamilies had been extensively modulated by gene duplications, deletions, and gene conversions around the time of the evolutionary split of the mouse and rat lineages, consistent with the hypothesis that V1R repertoires might be involved in reinforcing speciation events. Here, we generated genome sequence for one large cluster containing two V1R subfamilies in Mus spretus, a closely related and sympatric species to Mus musculus, and investigated evolutionary change in these repertoires along the two mouse lineages.

**Results:**

We describe a comparison of spretus and musculus with respect to genome organization and synteny, as well as V1R gene content and phylogeny, with reference to previous observations made between mouse and rat. Unlike the mouse-rat comparisons, synteny seems to be largely conserved between the two mouse species. Disruption of local synteny is generally associated with differences in repeat content, although these differences appear to arise more from deletion than new integrations. Even though unambiguous V1R orthology is evident, we observe dynamic modulation of the functional repertoires, with two of seven *V1Rb *and one of eleven *V1Ra *genes lost in spretus, two *V1Ra *genes becoming pseudogenes in musculus, two additional orthologous pairs apparently subject to strong adaptive selection, and another divergent orthologous pair that apparently was subjected to gene conversion.

**Conclusion:**

Therefore, eight of the 18 (~44%) presumptive *V1Ra/V1Rb *genes in the musculus-spretus ancestor appear to have undergone functional modulation since these two species diverged. As compared to the rat-mouse split, where modulation is evident by independent expansions of these two V1R subfamilies, divergence between musculus and spretus has arisen more by mutations within coding sequences. These results support the hypothesis that adaptive changes in functional V1R repertoires contribute to the delineation of very closely related species.

## Background

Pheromones are secreted chemicals recognized by members of the same species (conspecifics) that convey information about individual members. The vomeronasal organ (VNO) of terrestrial vertebrates is responsible for pheromonal responses that evoke social and reproductive behaviors, including male territorial aggression, sexual preference, and sexual maturity (reviewed in [1]). These responses are thought to be mediated by at least two unrelated gene families, referred to as V1Rs [2] and V2Rs [3-5], encoding G protein-coupled receptors (GPCRs) that are expressed on surfaces of sensory neurons in the VNO.

In addition to being the principle chemoreceptors in the olfactory and gustatory sensory systems, the large GPCR superfamily has prominent roles in transducing signals for diverse ligands, including ions, hormones, neurotransmitters, nucleotides, and photons [6]. The GPCR repertoire has a common seven transmembrane structure, and mammalian GPCR proteins are classified into six major families: the peptide-binding secretin types, the adhesion types that contain N-terminal domains with motifs implicated in cell adhesion functions (e.g., *EGF*-like repeats), the glutamate types (including *TAS1 *taste receptors and the V2Rs expressed in the VNO), the *frizzled *types, the Taste2 types (*TAS2 *taste receptors), and the large set of rhodopsin types (including the olfactory receptors) [6]. V1Rs share a distant relationship with the Taste2- and rhodopsin-types of GPCRs that bind ligands within transmembrane cavities (and that lack the large N-terminal binding domains characteristic of other GPCRs).

V1R gene repertoires vary significantly among species. In mouse and rat, there are a total of approximately 150 V1R genes from 12 phylogenetically distinct subfamilies [7,8]; members of each subfamily tend to be clustered at one or two chromosomal locations, reflective of a recent history of expansion by tandem duplications. In human and chimpanzee, the V1R repertoire consists almost entirely of non-functional pseudogenes, consistent with the loss of VNO function possibly concurrent with the advent of trichromatic color vision during primate evolution [8]. Surprisingly, the dog genome encodes only five intact V1R genes [8], the cow and opossum genomes encode less than a third the number of V1Rs as mouse [9], and several other mammalian species (such as pig [10], sheep [11], and ferret [12]) seem to similarly exhibit reduced VNO function as compared to rodents. Therefore, rodents appear to be exceptional with respect to their reliance on the VNO and V1R gene repertoires for mediating pheromone responses.

The mouse and rat V1R repertoires have modulated significantly since these two species diverged. In general, orthologous relationships between genes and syntenic relationships between clusters are difficult to map as a result of lineage-specific duplications and deletions. Two striking examples of delineation between the two species are the complete deletion of two subfamilies (*V1Rh *and *V1Ri*) in rat that represent the largest gene cluster in mouse, and the independent generation of two subfamilies (*V1Ra *and *V1Rb*) in both mouse and rat from a small number of ancestral genes in the presumptive ancestor [7,8,13]. Moreover, the *V1Ra/V1Rb *duplications in both species appear to have occurred over a very short period of evolution just following mouse-rat speciation, probably driven in part by a wave of LINE repeat integration within the locus [13]. These observations suggest that adaptive modulation of V1R repertoires were important in the reinforcement of interspecies communication barriers in the period immediately following speciation.

Unlike the odorant receptors of the main olfactory system, which exhibit ligand promiscuity as part of a combinatorial system of odor recognition (reviewed in [14]), the V1Rs of the VNO might respond to a very narrow range of ligands, if not exclusively to one ligand [15]. Moreover, while odorant receptor sensory neurons of the main olfactory system exhibit responsiveness in a concentration-dependent manner, the subset of vomeronasal sensory neurons responding to a particular stimulus do not change with the concentration of that stimulus. These observations suggest that different members of a V1R subfamily detect highly specific ligands, implying that modulation of subfamily repertoires could modulate physiologically distinct functions.

Little is currently known about V1R function. A recent knockout of the entire *V1Ra-V1Rb *cluster in mouse perturbed normal patterns of female aggression and caused male mice to show reduced sexual behavior towards females [16]. Similarly broad effects in sexual and social behaviors are evident in mice genetically deficient in *Trp2*, a protein required for vomeronasal neuronal signalling [17]. The low resolution of these phenotypes and the apparent phenotypic overlap between mice deficient in a subset of V1Rs and those fully deficient in V1R response suggest that individual V1Rs make combinatorial or additive contributions to broad behavioural patterns, as opposed to each V1R directing a distinct behaviour. To our knowledge, the hypothesis that V1Rs contribute to the establishment of mating barriers between species has not yet been investigated.

It is presumed that one function of pheromones is the establishment of prezygotic mating barriers so that unproductive mating is not attempted (reviewed in [18]). Prezygotic mating barriers can quickly arise to prevent non-productive interbreeding attempts between subpopulations otherwise capable of mating [19], and therefore, modulation of pheromones and their receptors might be especially important for selective breeding within sympatric populations. For example, the Mediterranean short-tailed mouse, Mus spretus, is sympatric with certain subspecies of Mus musculus in parts of southern Europe and North Africa. No hybrids of these two species have been observed in nature, suggesting that they will not interbreed (presumably due to a selective disadvantage in hybrid offspring), even though they can produce viable offspring in the laboratory [20]. These two species diverged approximately 1.1 million years ago [21,22]; with an estimated neutral substitution rate of ~1% per million years [23-25], we expect that typical orthologs will be ~98% identical between the two species.

In this study, we compare *V1Ra *and *V1Rb *gene repertoires in Mus spretus and Mus musculus in order to investigate whether, even over these very short evolutionary periods and in a background of very high sequence identity, these V1R repertoires exhibit the dynamic functional modulation observed between mouse and rat. Our results indicate that functional modulation of rodent V1R repertoires has occurred, albeit by diverse evolutionary paths, supporting the hypothesis that adaptive changes in these V1R repertoires have contributed to the delineation of even very closely related species.

## Results and Discussion

We produced a mixture of Mus musculus V1R probes composed of 200–300 bp PCR products from three members of the *V1Ra *and four members of the *V1Rb *subfamily (Table 1). We confirmed probe specificity by conducting a Southern blotting of musculus BAC clones known to contain V1R sequences from the *V1Ra*, *V1Rb*, *V1Rc*, *V1Rh*, and *V1Ri *subfamilies; only clones containing *V1Ra/V1Rb *sequences hybridized to our probes (not shown). We then used these *V1Ra/V1Rb *probes to screen the *CHORI-35 SPRET/Ei *BAC library . We identified 17 positive clones; we expected to identify approximately 12 positive clones given the redundancy of the library (three-fold) and the anticipated size of the *V1Ra/V1Rb *cluster (~700 kb, or approximately four BAC-sized inserts in length). We sequenced the ends of all 17 BACs and confirmed that all non-repeat BAC-end sequences were unambiguously orthologous to the *V1Ra/V1Rb *locus in the most recent musculus genome assembly (UCSC Genome Browser, July 2007). These BAC-end sequences, along with an independent Southern blot restriction fragment analysis of the isolated clones, were used to select a minimal tiling path of four BAC clones that we thought would span the entire Spretus *V1Ra/V1Rb *cluster, with the two flanking BACs extending out to at least one non-V1R gene at either end of the cluster. These four BACs were then subjected to shotgun sequencing, yielding near 'comparative-grade' finished sequence as described in Blakesley *et al*. [26] (Genbank accession numbers: AC225052, AC225271, AC225873, AC229624).

**Table 1:** Mus musculus probes used to screen the Mus spretus BAC library.

Probe	Size (bp)	>90% Homology
MUS.A6	297	A1,A2,A3,A4,A5,A6
MUS.A7	297	A2,A7,A8
MUS.A9	297	A9
MUS.B1	222	B1
MUS.B4	222	B2,B4,A2
MUS.B7	222	B7,B9
MUS.B8	222	B8

Probes of the indicated size were generated by PCR using gene-specific primers. A list of musculus V1R gene sequences with at least 90% nucleotide identity to each probe illustrates that the probe mixture should efficiently hybridize to BAC clones containing any of the entire musculus *V1Ra/V1Rb *repertoire.

### Production of a Mus spretus sequence assembly reveals a conserved synteny map

**Figure 1:**
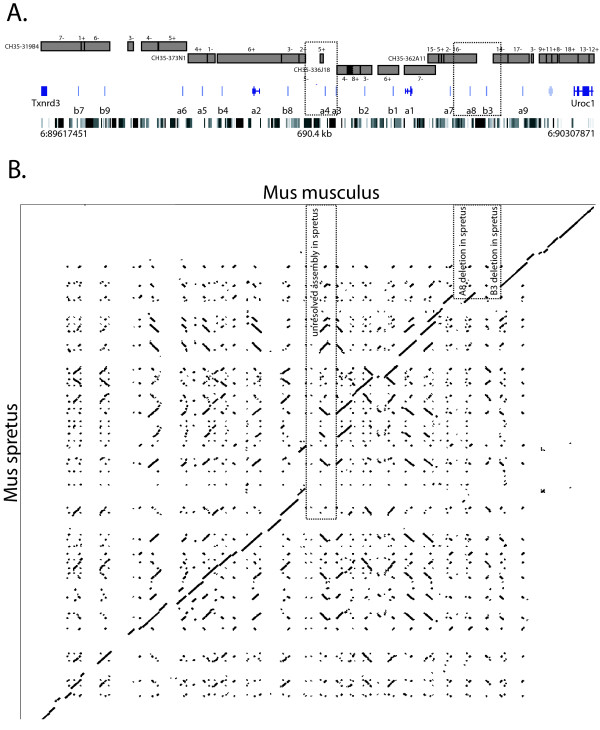
**Synteny map for Mus spretus versus Mus musculus**. **A**. Map of the 690.4-kb Mus musculus *V1Ra/V1Rb *gene cluster on chromosome 6 (89617451–90307871 in July, 2007 assembly, UCSC Genome Browser) showing flanking non-V1R genes (*Txnrd3*, *Uroc1*) and the 16 intact V1R genes in the cluster. LINE repeats (black stripes) are shown below gene annotations. The locus assembly in Mus spretus is shown above the musculus map, with the ordered contigs indicated by rectangles (contig numbers and orientations above/below rectangles). The total spretus assembly is ~716.5 kb. Dotted boxed regions indicate the unresolved spretus assembly corresponding to the *V1ra3-V1ra4 *region in musculus and the presumed deleted segment in spretus corresponding to the *V1ra8-V1rb3 *region in musculus. The contiguity of the spretus-musculus assemblies are illustrated by the diagonal in the dot matrix in panel **B**.

We mapped contigs from spretus BAC sequences onto the most recent musculus assembly using PipMaker . We generated a presumptive locus assembly of ordered/oriented spretus contigs that maximizes contiguous alignment between the two species (Fig. 1). Chained alignments between musculus and spretus sequences encompass ~485 kb, or approximately 68% of the spretus assembly and 67% of the musculus assembly. The remaining portion of the two mouse sequences that cannot be unambiguously aligned consists predominantly of repeats that have integrated or deleted in one but not the other lineage (see following section). It is possible that this presumptive assembly is incorrect if there have been additional evolutionary events that have disrupted local synteny. However, we note that the resulting spretus sequence produces an array of putative orthologous V1R genes that exactly matches the order and orientation observed in musculus.

**Figure 2:**
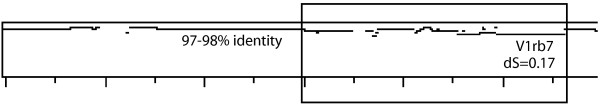
**Unexpected low orthology in the vicinity of the *V1rb7 *gene**. PipMaker plot with chained alignments illustrating the contiguous high level of orthology (97–98%) evident upstream and downstream of the musculus *V1rb7 *gene. Hash marks indicate 1-kb intervals. The lower level of more disrupted synteny is confined to the region encompassing the musculus *V1rb7*/spretus *YUA.5 *orthologs, whose synonymous substitution rate (dS) is ~17%, or approximately 8.5-fold more diverged than expected. This gene pair might have been subject to gene conversion in one or both lineages.

The synteny map shown in Figure 1 reveals three ambiguities. First, the spretus sequences at one end of the cluster have subregions that exhibit lower than expected levels of orthology. As noted, neutral sequences are expected to diverge ~2% between the musculus-spretus split, and yet, orthologous sequences in the vicinity of the musculus *V1rb7 *gene, as well as other subregions (e.g., in the vicinity of the spretus *YUA.6pg*/musculus *MUSpg.89648 *orthologs), exhibit unexpectedly high divergence (Fig. 2). The *V1rb7-YUA.5 *are closest mutual homologs in the same relative position and orientation in their respective clusters, and therefore are putative orthologs, yet exhibit a synonymous substitution rate (dS) of ~17%, which is much greater than should be the case in the roughly one million years since the two Mus species diverged. It seems likely that these subregions have been subject to gene conversion events in one or both species that have obscured what is otherwise a much closer relationship. Nevertheless, this high level of sequence divergence might underlie divergent functions between these orthologs.

Second, the spretus locus appears to have deleted a segment in the vicinity of the musculus *V1ra8-V1rb3 *genes, although it is possible the segment is missing at this phase of sequence completion (Fig. 1). In musculus, the similar *V1ra7 *and *V1ra8 *paralogs exhibit a dS = ~6.5%, consistent with a duplication event that occurred prior to the musculus-spretus split. Therefore, the absence of *V1ra8 *in the spretus assembly is more consistent with loss in spretus than gain in musculus. The fact that *V1rb3*, the next gene in the musculus cluster, is also missing in spretus reinforces the hypothesis that a segmental deletion that includes both *V1ra8 *and *V1rb3 *occurred in the spretus lineage.

Third, the spretus locus is unresolved in the vicinity of the musculus *V1ra3-V1ra4 *paralogs (Fig. 1). This region is near the junction of the two middle BACs (*CH35-373N1 *and *CH35-336J18*). There is no apparent overlap in these two BACs (i.e., with 100% sequence identity), although both BACs have highly similar subsequences as a consequence of each having possible orthologs to *V1ra3/V1ra4 *(*YUB.3 *and *YUC.6*, respectively). dS values predict that paralagous duplications in this region occurred prior to the musculus-spretus split (dS for *V1ra3-V1ra4 *= ~6%; dS for *YUB.3-YUC.6 *= ~5%), and yet the dS values of cross-species comparisons are greater than expected for orthology (dS range = ~4–7%). Therefore, the evolutionary history relating these four homologs might again be obscured by lineage-specific gene conversions. We also note that no putative ortholog to the *YUC.7pg *V1R pseudogene (most similar to musculus *V1rb4*), located just downstream of *YUC.6 *on *CH35-336J18*, is present in the musculus assembly, possibly indicating that lineage-specific rearrangements might have disrupted synteny in this region. Further resolution of this phylogeny would require higher-quality and more complete sequence across this region, so that the full V1R content/positioning and surrounding non-coding gene blocks through this region could be more rigorously analyzed.

### There is a low level of lineage-specific LINE integration

**Figure 3:**
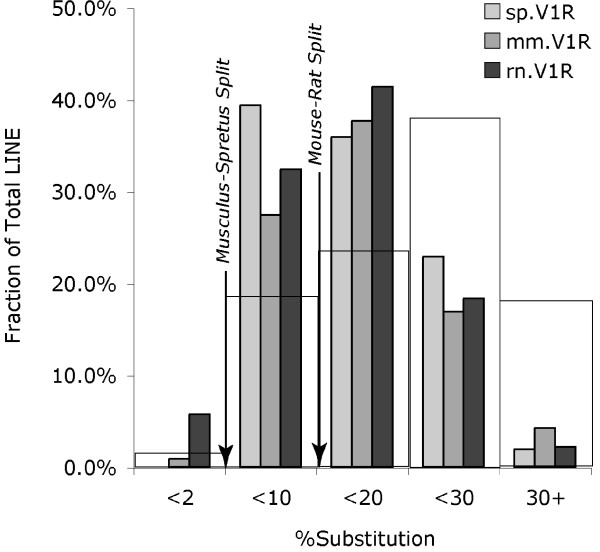
**Comparison of LINE ages at the three rodent *V1Ra/V1Rb *clusters to the mouse genome at large**. The percentage of total LINE repeat content likely to be lineage-specific with respect to the musculus-spretus split (annotated in RepeatMasker with <2% substitution) or with respect to the mouse-rat split (annotated in RepeatMasker with <10% substitution), as well as older LINE content (>20% substitution), is compared between Mus spretus (sp.V1R, light gray), Mus musculus (mm.V1R, dark gray), and rat (rn.V1R, black), as well as to the Mus musculus genome at large (wider, open rectangles). The assumption that LINE repeats integrating since the mouse-rat split would exhibit <10% substitution is based on an estimate of neutral substitution levels of <20% among orthologous sequences (20% substitution between orthologs = 10% substitution along both lineages since the ancestral node). These data indicate that the majority of dense populations of LINE repeats at these rodent V1R loci integrated around the time of the mouse-rat split (but not since the musculus-spretus split) and that rodent V1R loci have younger LINE repeat content as compared to the genome at large.

We previously observed that the *V1Ra *and *V1Rb *subfamilies underwent a burst of duplications independently in both the mouse and rat lineage shortly after these two species diverged [13]. The timing of these gene duplications was correlated with the timing of LINE repeat integrations at this locus, possibly suggesting a cause-effect relationship. As in musculus and rat, LINE repeats are abundant within the spretus *V1Ra/V1Rb *locus, comprising ~39% of the sequence and clearly demarking this V1R gene cluster from surrounding LINE-poor non-V1R territory (Fig. 1; Table 2). As noted above, a significant fraction of the broken synteny between musculus and spretus is LINE content present in one but not the other lineage. However, unlike with mouse versus rat, where lineage-specific LINE integration accounted for the vast majority of broken synteny, we find very little evidence for lineage-specific LINE integration since the spretus-musculus split (Fig. 3; Table 2). Therefore, the LINE (and other repeat) blocks present in one but not the other Mus species are more likely due to lineage-specific deletion, recombination, and/or gene conversion of pre-existing LINE content.

**Table 2:** Repeat content within mouse V1R loci.

	musculus	spretus
GC%	42.0%	40.6%
Repeats	60.6%	59.9%
LINE	39.5%	39.3%
**LINE < 9.5**	**62.8%**	**61.8%**
**LINE < 2.0**	**1.9%**	**0.0%**
SINE	2.3%	2.3%
LTR	16.9%	16.3%
Other	1.9%	2.0%

The percent GC base composition (GC%), total repeat content (Repeats), LINE repeat content (LINEs), SINE repeat content (SINEs), LTR repeat content (LTRs), and other repeat types (Other) are shown for the ~721-kb musculus and ~717-kb spretus sequences encompassing the *V1Ra/V1Rb *gene cluster. The fraction of the total LINE repeat content annotated by RepeatMasker with <9.5% substitution (mouse lineage-specific with respect to rat) and <2.0% substitution (musculus/spretus lineage-specific) is bolded.

### Species-specific modulation of functional orthologs

**Figure 4:**
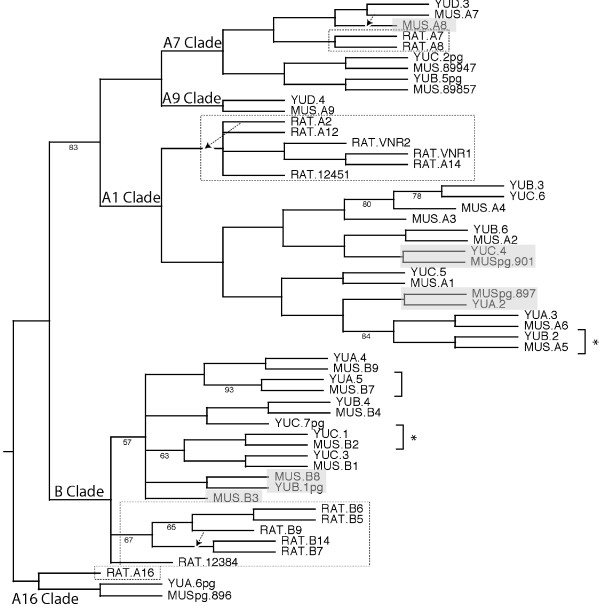
**V1R gene tree**. Distance tree produced using 56 codon-aligned V1R sequences (906 nucleotides length) from mouse (musculus V1Rs denoted with "MUS" prefix; spretus V1Rs denoted with "YU" prefix) and rat ("RAT" prefix; dotted boxed clades). Pseudogenes are denoted by "pg" in their names. Musculus-spretus orthologous pairs in which one species encodes an intact V1R and the other species encodes an apparent pseudogene are shaded. Deleted V1Rs in one species but not the other are also shaded. Brackets with asterisks denote the two orthologous gene pairs apparently subject to adaptive selection (dS/dN < 1; see text), and the bracket without asterisk denotes the one orthologous gene pair apparently subject to gene conversion (Fig. 2). From this phylogeny, we infer that the rodent ancestor had five V1R genes: a homolog to rat *A16*, a homolog to mouse *A1*, a homolog to mouse *A9*, a homolog to mouse *A7*, and a *B*-like homolog. Bootstrap values (1000 replicas) for nodes <95% are indicated. Dashed lines with arrows show different locations of the *MUS.A7*, *RAT.A2*, and *RAT.B9 *branches in a parsimony bootstrap tree (1000 replicas) (not shown).

V1R orthologs are easily identified based on two criteria: first, orthologous pairs occupy the same relative position and orientation in the syntenic map (Fig. 1); and second, orthologous pairs exhibit synonymous substitution (dS) rates approximating the expected ~2% in codon-aligned sequences (Table 3). Noted exceptions with conserved synteny but with higher than expected dS levels (the *YUA.5-MUS.B7 *and *YUA.6pg-MUSpg.89648 *putative orthologous pairs) and where orthologous relationships are ambiguous (the *YUB.3-YUC.6-MUS.A3-MUS.A4 *orthologous groups) are discussed separately below. A phylogenetic tree of spretus V1R genes and pseudogenes that includes members of the *V1Ra *and *V1Rb *subfamilies from musculus and rat illustrates these orthologous relationships (Fig. 4).

**Table 3:** Putative orthologous V1R gene pairs identified in the two Mus species.

musculus	spretus	dS	dS/dN
***MUSpg.89648***	***YUA.6pg***	**0.06**	0.94
**MUS.B7**	**YUA.5**	**0.17**	3.04
MUS.B9	YUA.4	0.04	5.11
*MUSpg.89739*	*YUA.1pg*		
*MUSpg.89755*	YUA.2	0.04	1.70
MUS.A6	YUA.3	0.03	2.33
**MUS.A5**	**YUB.2**	0.01	**0.99**
MUS.B4	YUB.4	0.03	2.24
*MUSpg.89857*	*YUB.5pg*	0.04	1.27
MUS.A2	YUB.6	0.03	2.20
MUS.B8	*YUB.1pg*	0.02	2.08
*MUSpg.89947*	*YUC.2pg*	0.03	1.16
**MUS.A4**	**YUB.3**	**0.06**	2.59
**MUS.A3**	**YUC.6**	**0.07**	2.43
*(missing)*	*YUC.7pg*		
**MUS.B2**	**YUC.1**	0.01	**0.25**
MUS.B1	YUC.3	0.03	5.39
MUS.A1	YUC.5	0.03	17.68
*MUSpg.90103*	YUD.2	0.04	2.52
MUS.A7	YUD.3	0.03	1.40
MUS.A8	*(missing)*		
MUS.B3	*(missing)*		
*MUSpg.90192*	YUD.1pg		
MUS.A9	YUD.4	0.02	1.17

Putative orthologs for Mus musculus and Mus spretus are shown in the order they appear in their respective clusters. Italics indicate likely pseudogenes. Synonymous substitution rates (dS) for orthologous pairs are typically in the 2–4% range (exceptional pairs are bolded). A range of ratios for synonymous to non-synonymous substitution rates (dS/dN) are evident; two pairs possibly subject to adaptive selection with dS/dN < 1 are bolded.

This tree suggests that the rodent ancestral cluster probably had at least five V1R genes, four from the *V1Ra *subfamily (*V1ra1-like*, *V1ra7-like*, *V1ra9-like*, and *V1ra16-like*) and one from the *V1Rb *subfamily. The *V1ra9*-like ancestor at one end of the cluster has been deleted in rat, and the *V1ra16*-like ancestor at the other end of the cluster became a pseudogene in mouse. The three other ancestral genes (the *V1Rb-like*, *V1Ra1-like*, and the *V1ra7-like *ancestors) duplicated independently in the mouse and rat lineages (Fig. 4). Therefore, the functional repertoire is significantly different between mouse and rat, driven by lineage-specific mutations, deletions and duplications.

We do not identify any lineage-specific V1R duplications since the musculus-spretus split. The *YUD.3-MUS.A7-MUS.A8 *clade in Figure 4 shows an apparent musculus-specific duplication, however as noted previously, the synonymous substitution rate (dS) between the *A7-A8 *pair is ~6.5%, or much greater than ~2% expected for the spretus-musculus split, and thus, the paralogous duplication probably occurred before the two mouse species diverged. Therefore, this clade is better explained by spretus-specific gene deletion of the *A8 *homolog (assuming it is not missing within gaps of the unfinished spretus sequence). In addition, the *YUB.3-YUC.6-MUS.A4-MUS.A3 *clade (Figure 4) also shows apparent species-specific duplications, however as noted previously, neither the spretus pair (*YUB.3-YUC.6*, dS = 5%) nor the musculus pair (*MUS.A4-MUS.A3*, dS = 6%) exhibit a low enough synonymous substitution rate to be consistent with post-speciation duplication. Instead, it seems more likely that a paralogous duplication occurred prior to the musculus-spretus split, and that the unexpected topology of this clade is due to noise in the analysis or to gene conversion events that might have obscured relationships. Therefore, in contrast to mouse and rat, the musculus-spretus *V1Ra/V1Rb *repertoires have not significantly diverged by lineage-specific gene duplications, a result that seems consistent with the observed low incidence of lineage-specific repeat activity.

Instead, divergence of *V1Ra/V1Rb *repertoires between spretus and musculus is evident by selective loss of function (Table 3). Of the eight orthologs in the *V1ra1-like *clade, two have apparently become pseudogenes in musculus. Of the three orthologs in the *V1ra7-like-V1ra9-like *clade, one has apparently deleted in spretus. And of the seven orthologs in the *V1Rb *clade, one has deleted and another has become a pseudogene in spretus. Therefore, of the 18 *V1Ra/V1Rb *genes presumed to be functional in the common Mus ancestor, five (~28%) have lost function in one but not the other Mus lineage.

We emphasize however, that additional V1R sequences could reside within the remaining gaps in the current spretus sequence assembly. We compared *in silico *digests of the BAC sequences by three separate restriction endonucleases (*BamH1*, *EcoR1*, and *HindIII*) to the same generated in the laboratory and imaged on a high resolution gel electropherogram. This comparison of sequence to physical data allows a validation of sequence assembly (order and orientation) for each BAC. From this analysis, we estimate that all gaps internal to each BAC assembly are <2 kb (not shown), too small to be expected to contain additional V1R gene blocks (~5–10 kb in size). It is also unlikely that a V1R gene resides between the leftmost two BACs (*CH35-319B4 *and *CH35-373N1*) in the spretus assembly, since based on mapping to the musculus assembly, the missing inter-BAC region is only ~3.5 kb and this region does not contain a V1R gene in musculus. In contrast, the unresolved region between the two middle BACs (*CH35-373N1 *and *CH35-336J18*) might be >40 kb and is likely to contain several V1Rs. In particular, identification of the complete set of the "A3-like" and "A4-like" homologs in spretus, and their evolutionary relationship with *V1ra3 *and *V1ra4 *in musculus, will require the identification and analysis of a genomic DNA clone that spans this gap in our assembly. Thus, it remains possible that additional functional divergence between spretus and musculus will be revealed by future sequence from this region.

### Evidence for positive selection among orthologous pairs

It has been previously noted that rodent V1R homologs exhibit unusually low dS/dN ratios as compared to typical gene pairs in these species [27], suggestive of a tendency for adaptive selection, presumably to meet changing niche- and species-specific requirements. Previous studies have focused on mouse paralogs, rat paralogs, and mouse-rat homologs, but not mouse-rat orthology, because orthologs cannot be unambiguously assigned due to excessive lineage-specific duplications that obscure orthologous relationships. In the current study, we specifically investigated selective pressures acting on putative functional orthologs, an investigation made possible by the unambiguous assignment of orthologous gene pairs.

We observe that two orthologous pairs, *YUB.2-MUS.A5 *(dS/dN = 0.99) and *YUC.1-MUS.B2 *(dS/dN = 0.25), exhibit greater non-synonymous than synonymous substitution rates (Table 3). This observation is consistent with adaptive selection acting on these pairs since the musculus-spretus split. Selection for new amino acids in these V1R proteins could indicate divergence from ancestral functions, and therefore, these V1Rs might contribute to additional functional delineation between the two species. It is important to note that with so few nucleotide substitutions accumulated between these orthologs (9 and 15 mutations, respectively), dS/dN ratios can be misleading due to small sample sizes. For example, we cannot assert that the dS/dN ratio for the *YUB.2-MUS.A5 *pair is significantly different than neutrality (dS/dN = 1), a possibility if one or both genes have become cryptic pseudogenes (i.e., non-functional as opposed to diverged function). The argument for adaptive selection between *YUC.1-MUS.B2 *is much more compelling, where 14 of 15 observed mutations have caused amino acid changes.

In order to assess the possible biological significance of amino acid substitutions observed in these two V1R gene pairs, we mapped mutations onto predicted protein structures. We compared these locations to other gene pairs presumed to be under purifying selection (six orthologous pairs with dS/dN > 2) (Table 4). Non-synonymous mutations in the six V1R pairs presumed to be under purifying selection have accumulated more frequently within *transmembrane domain-4 *(TM4), TM6, and the intracellular loop between TM5 and TM6. Non-synonymous mutations in YUB.2-MUS.A5 and YUC.1-MUS.B2, the two V1R pairs postulated to be under positive selection, also exhibit a bias for accumulation within the intracellular loop between TM5 and TM6, but exhibit a greater incidence of amino acid change in TM3 and TM1. Three non-synonymous mutations (3/44 opportunities = ~7% incidence) have accumulated within TM3 (two for YUB2-MUSA5 and one for YUC1-MUSB2), whereas no amino acid substitutions are observed in this transmembrane domain among the other intact genes (0/132 opportunities = 0% incidence). Five non-synonymous mutations (5/24 opportunities = ~21% incidence) have accumulated within TM1 (one for YUB2-MUSA5 and four for YUC1-MUSB2), whereas only one amino acid substitution is observed in this transmembrane domain among the other intact genes (1/72 opportunities = ~1% incidence). Interestingly, previous analysis of mouse-rat gene pairs indicated a paucity of non-synonymous mutations within TM1 and TM3 [28], raising the possibility that different regions of the V1R structure adapted during the mouse-rat split than during the musculus-spretus split.

**Table 4:** Distribution of non-synonymous mutations within the predicted V1R protein structure.

Domain	Conserved (21)	dS/dN > 2 (6)	dS/dN < 1 (2)
E1 (23)		0.72	2.17
TM1 (12)	G24,N28,L31	1.39	**20.83**
I1 (14)		2.38	0.00
TM2 (22)	L60,L63	1.52	0.00
E2 (25)	D72,W80,C85,R96	1.33	2.00
TM3 (22)	L106,L114	0.00	**6.82**
I2 (13)	K125	1.28	0.00
TM4 (23)	Y143	**5.07**	2.17
E3 (39)	N159,C172,R192	0.85	2.56
TM5 (18)	L211	0.93	2.78
I3 (26)	L221,S227,A236	**5.77**	**9.62**
TM6 (20)		**4.17**	2.50
E4 (17)	R261	0.98	**8.82**
TM7 (16)		1.04	0.00

The size (number of amino acid residues, brackets) of predicted domains structures (E = extracellular, I = intracellular, TM = transmembrane) and the locations of the 21 most conserved amino-acid residues in rodent V1R proteins are shown. The incidence of non-synonymous mutations in six orthologous gene pairs (*MUS.A6-YUA.3*, *MUS.B9-YUA.4*, *MUS.B4-YUB.4*, *MUS.A2-YUB.6*, *MUS.B1-YUC.3*, and *MUS.A1-YUC.5*) with dS/dN > 2 (presumed purifying selection) were compared to two orthologous gene pairs (*MUS.A5-YUB.2 *and *MUS.B2-YUC.1*) with dS/dN < 1 (presumed adaptive selection). Numbers shown are the incidence of occurrence (observed percent mutations/opportunities) within a particular structural domain (outliers are bolded).

At higher resolution, we observe an interesting mutational trend for YUB.2-MUS.A5 and YUC.1-MUS.B2: a tendency to accumulate non-synonymous substitutions immediately adjacent to the most universally conserved amino acid residues (Table 4). Assuming a random distribution, we would expect a 21% incidence of amino acid changes immediately adjacent (+/- 1 amino acid position) to the 21 most well-conserved residues (see Methods). This expectation is slightly greater than what we observe for six gene pairs presumed to be evolving under purifying selection (6/36 amino acid changes = 17%). In contrast, 35% (8/23) of the amino acid changes in YUB.2-MUS.A5 and YUC.1-MUS.B2 occur immediately adjacent to these well-conserved residues. Note that only in one of these eight cases did the well-conserved amino acid itself change, therefore this bias would appear to have more to do with modulating rather than eliminating the function of these conserved residues. Analysis of a larger sample size when additional Mus spretus V1R sequences become available are required to further evaluate the significance of these observations, and elucidation of the physiological significance of these amino acid changes awaits structure-function and ligand-binding studies.

## Conclusion

We have generated ~700 kb of genome sequence encompassing a V1R putative pheromone receptor locus (*V1Ra *and *V1Rb *subfamilies) in Mus spretus. The genome features of this locus resemble the syntenic locus in Mus musculus, including a comparable number of V1R genes with little evidence for gene duplication/deletion, organized in a comparably sized region flanked by the same non-V1R genes on either side. These two mouse V1R loci also exhibit a similar high density of LINE repeat content that appears to have predominantly integrated just before and since the mouse-rat split. Although disruptions in synteny are largely accounted for by the presence/absence of LINE repeat content in one or the other lineage, these LINEs appear to be too old to have integrated by retrotransposition since the musculus-spretus split, and thus, seem to have either arrived by other mechanisms or been lost in one of the lineages. Therefore, LINE integrations and V1R gene birth/death processes are not characteristics of the divergence between musculus and spretus, in contrast to the previous observations made for mouse and rat. Instead, functional V1R repertoires in musculus and spretus appear to have diverged by adaptive evolution in which complementary V1R subsets have deleted along the two lineages and specific orthologs appear to be subject to diversifying selection and gene conversion. While the evolutionary paths differ, the outcome is similar among mouse and rat species: nearly half of the mouse V1R repertoire (8/18) have been subject to dynamic modulation, consistent with the hypothesis that even very closely related species, such as Mus musculus and Mus spretus that are separated by roughly ~ one million years, have evolved species-specific chemosensory functions.

## Methods

### Probe Production and Library Screening

A panel of mouse V1R probes was selected that would together hybridize to all V1R genes in the Mus musculus *V1Ra/V1Rb *cluster on chromosome 6, assuming hybridization required >90% nucleotide identity (Table 1). Gene-specific PCR primers were used to amplify 200–300 bp from each gene; PCR was conducted using digoxigenin- (Dig-) labeled dUTP (Roche). Probes were mixed for subsequent medium-high stringency hybridization to genomic filters of an arrayed Mus spretus BAC library (CHORI-35 SPRET/Ei BAC library, Children's Hospital Oakland Research Institute). Glycerol stocks were prepared for positive clones, and BAC DNA was isolated using the Qiagen Midi Prep kit (Qiagen).

### BAC Mapping

BAC ends were sequenced using proprietary protocols by Agencourt Bioscience QuickLane Sequencing service using the SP6 and T7 priming sites on the pTARBAC2.1 vector DNA. Resulting BAC-end sequences were BLAT-matched onto the Mus musculus sequence assembly (UCSC Genome Browser, July 2007) in order to provide initial mapping information, assuming that synteny was conserved between the two closely related Mus species. In addition, EcoR1-digested BAC DNA was Southern blotted and hybridized with the above V1R probe mixture to estimate the number of V1R genes per BAC and to provide additional mapping resolution. From these analyses, four BAC clones (*CH35-319B4*, *CH35-373N1*, *CH35-336J18*, *CH35-362A11*) that appeared to be an efficient tiling path spanning the entire V1R cluster were selected for sequencing.

### BAC Sequencing

The selected BACs were subjected to shotgun sequencing using standard methods, resulting in 7, 6, 8, and 18 contigs in the four BAC assemblies, respectively. These assemblies were subjected to near 'comparative-grade' sequence finishing as described in Blakesley *et al*. [26]. A majority of contigs were ordered and oriented by read pairs of gap spanning subclones. Other contigs were established by alignment to the reference (M. musculus) sequence (see below). Contig maps were verified by comparing restriction digest patterns from laboratory produced gels to those generated *in silico *from the consensus sequences. The four BAC sequences are available in Genbank with the following accession numbers (*gi *numbers indicate draft versions used in this analysis): AC225052 (gi:189095727), AC225271 (gi:183227749), AC225873 (gi:187960227), AC229624 (gi:192807370).

### Synteny Map

The *CH35-319B4 *BAC sequence was assembled into seven contigs that include the ortholog to the *Txnrd3 *gene that flanks the "left" end of the *V1Ra/V1Rb *locus in the Mus musculus assembly, as well as putative orthologs to *V1rb7*, *V1rb9*, and *V1Ra6*, the three "left"-most V1Rs in this assembly. The *CH35-373N1 *BAC sequence was assembled into six contigs that include putative orthologs to *V1ra5*, *V1rb4*, *V1ra2*, *V1rb8*, and *V1ra4*, consistent with synteny to the next segment (moving "right") in the musculus assembly. The *CH35-336J18 *BAC sequence was assembled into eight contigs that include putative orthologs to *V1Ra3, V1rb2, V1rb1*, and *V1ra1*, consistent with sytneny to the next segment (moving "right") in the musculus assembly. The *CH35-362A11 *BAC sequence was assembled into 18 contigs that include putative orthologs to *V1ra7 *and *V1ra9*, two "rightmost" V1Rs in the musculus cluster, as well as the ortholog to the *Uroc1 *gene that flanks the "right" end of the musculus locus. We confirmed a ~9.5 kb overlap of the *CH35-336J18 *and *CH35-362A11 *BACs (the two "rightmost" BACs), but cannot confirm overlap for the other two junctions. Based on synteny mapping, we estimate that the gap between CH35-319B4 and CH35-373N1 (the two "leftmost" BACs) is approximately 3.5 kb, a region not predicted to contain V1R-like sequence. The junction between the two middle BACs, *CH35-373N1 *and *CH35-336J18*, is less resolved. As indicated, the former BAC contains a putative ortholog to *V1rb8*, and the latter BAC contains a putative ortholog to *V1rb2 *(located ~95 kb to the "right" of *V1rb8*), and both BACs contain putative orthologs to the similar *V1ra3 *and *V1ra4 *gene pair located between *V1rb8 *and *V1rb2 *in the musculus assembly (Fig. 1). However, our phylogenetic and neutral substitution analysis (see text) indicates that there might have been lineage-specific gene conversions or recombinations involving the *V1ra3-V1ra4 *region, and therefore, it is not possible to unambiguously map synteny where these two middle BACs intersect. Contigs were assembled and oriented for each spretus BAC in order to maximize contiguous alignment with the musculus assembly; short contigs without unambiguous alignment to musculus were omitted from this "locus assembly". The resulting spretus sequence assembly was analyzed for repeat content using the *RepeatMasker *algorithm (; Institute for Systems Biology, Seattle).

### V1R gene analysis

All spretus contigs (including short contigs excluded from the "locus assembly") were surveyed for V1R gene content using a virtual V1R probe designed from a composite of all known musculus V1R sequences. A total of 22 V1R-like sequences were identified, including 15 intact V1Rs that encode open reading frames. The 15 intact V1Rs, along with five of the pseudogenes, could be confidently aligned over a 906-bp stretch. One of the excluded pseudogenes, *YUA.1pg *(located between orthologs to *V1rb9 *and *V1ra6 *on *CH35-319B4*), is located at a syntenic position to a pseudogene in musculus located at the 89739 kb position on chromosome 6 (UCSC Genome Browser, July 2007 assembly). The other pseudogene excluded from the alignment, *YUD.1 pg *(located between orthologs to *V1ra7 *and *V1ra9 *on *CH35-362A11*), is located at a syntenic position to a pseudogene in musculus located at the 90192 kb position on chromosome 6. We note that the spretus *YUC.4 V1R *was present in the overlapping sequence of both the *CH35-362A11 *and *CH35-336J18 *BAC assemblies. We produced nucleotide alignments derived from amino acid alignments of the 20 V1R sequences ("codon-aligned"; see Additional file 1), as well as homologous sequences from musculus and rat. We used the *SNAP *on-line tool  to analyze synonymous and non-synonymous substitution histories, and the *Paup *program (Sinauer Associates) for phylogenetic reconstructions.

## Authors' contributions

VCK designed and produced V1R probes, screened and isolated clones from the spretus BAC library, and conducted Southern blots used to select BAC clones for subsequent sequencing. MG prepared BAC clones for sequencing and conducted sequence analyses. The NISC Comparative Sequencing Program performed shotgun sequencing of the BAC clones to produce high-quality sequence data; this included detailed sequencing finishing and confirmatory bioinformatics analyses to validate the accuracy of the generated sequence. EDG supervised the BAC sequencing process and contributed to the intellectual content of the manuscript. RPL conducted evolutionary analyses and wrote the manuscript.

## Supplementary Material

Additional file 1 **V1R gene alignments used in this study.** Click here for file
